# *Anopheles stephensi* in Africa requires a more integrated response

**DOI:** 10.1186/s12936-022-04197-4

**Published:** 2022-05-31

**Authors:** Abraham Mnzava, April C. Monroe, Fredros Okumu

**Affiliations:** 1 African Leaders Malaria Alliance, Dar es Salaam, Tanzania; 2 Johns Hopkins Center for Communication Programs, Baltimore, MA USA; 3 Ifakara Health Institute, Dar es Salaam, Tanzania

**Keywords:** *Anopheles stephensi*, Horn of Africa, Biological threats, Malaria, Integrated vector control, Vector surveillance

## Abstract

There are increasing reports of the Asian malaria mosquito, *Anopheles stephensi* invading and spreading in Eastern Africa. We discuss the importance of these invasions in the context of broader challenges facing malaria control in Africa and argue against addressing it as an isolated problem. *Anopheles stephensi* is only one of multiple biological threats facing malaria control in the region—and is itself an indication of wide-ranging weaknesses in vector surveillance and control programs. Expanded investigations are needed in both urban and rural areas, especially in countries serviced by the Indian Ocean trade routes, to establish the full extent and future trajectories of the problem. More importantly, instead of tackling this vector species as a stand-alone threat, affected countries should adopt more integrated and multi-sectorial initiatives that can sustainably drive and keep out malaria.

## Introduction

*Anopheles stephensi* was historically considered an Asian malaria vector and has been one of the major drivers of transmission in cities across India, Iran and Pakistan, as well as the Arabian Peninsula. It was detected in Africa for the first time in 2012, in the city of Djibouti [1]. Subsequent investigations have confirmed the presence of the vector species in multiple sites in Ethiopia [2], Somalia [3] and Sudan, where one of the latest discoveries happened serendipitously during an urban search for culicines [4]. More recent surveys in Sudan have confirmed extensive geographical spread of the vector, including observations in districts bordering six other countries without any prior evidence of the species [5].

Given these observations, there are now serious concerns that *An. stephensi* may already be present in, or is spreading to, areas outside of the Horn of Africa. While malaria in Africa has been overwhelmingly a rural disease, transmission could rise in urban areas where *An. stephensi* populations establish [6]. One geo-statistical model has predicted that the species could spread to many other African cities, eventually putting at least 126 million people at risk [7].

In 2019, the World Health Organization released a threat notice highlighting the spread of *An. stephensi* in the Horn of Africa; and warned public health authorities to be vigilant [8]. This alert, though issued seven years after the initial observations in Djibouti, has played an essential role in galvanizing efforts to address the threat. While there is now increasing awareness within the global malaria community, responses to date have largely been siloed and reactionary. In particular, the problem is by the scientific community as simply another research problem—thus ignoring the broader issues that most malaria control programs currently face.

This article positions *An. stephensi* within the broader context of biological threats to malaria control in Africa and discusses opportunities to better understand and sustainably address it.

## Broadening our lens

### Symptom of a broader weaknesses

The invasion and spread of *An. stephensi* in eastern Africa indicate broader weaknesses in vector surveillance and control. Many countries, including those affected by the invasion still do not have sufficient capacity and resources to carry out effective entomological assessments – including monitoring for invasive species, understanding the insecticide resistance profiles, or investigating pathogen transmission by prevailing vector populations [9, 10]. This is compounded by inadequate capacity to analyse and use local data for decision making, environmental changes and urbanization trends as well as the failures to institute multi-sectoral efforts, particularly the neglect of environmental sanitation as a key component in vector control. Some of these control programs are also unable to implement vector control interventions in a timely and comprehensive manner. In a recent global analysis, only 8% of the national malaria programs reported having sufficient capacity to implement vector surveillance; and only 28% had capacity to implement larval source management [9]. This study concluded that most countries will not defeat malaria without major investments to improve staffing and boost system-wide capacity [9].

### Anopheles stephensi and other biological threats

According to the WHO Malaria Threats Map [3], *An. stephensi* is one of several biological threats currently facing malaria control. The other important biological concerns include: (a) the widespread resistance of malaria vectors to public health insecticides, notably those used for insecticide treated nets (ITNs) and indoor residual spraying (IRS) [11]; (b) the emerging threat of parasite resistance to frontline malaria treatments [12, 13] and (c) the emergent failures of malaria rapid diagnostic tests (mRDTs) due to HRP2/3 gene deletions in *Plasmodium falciparum* [14]. In addition, health system disruptions caused by epidemics such as Ebola [15] and the COVID19 [16] pandemic can reverse malaria gains by derailing case management and prevention services. In the Horn of Africa region, where *An. stephensi* sightings are currently concentrated, the relatively high prevalence of *Plasmodium vivax* malaria further complicates the situation [17]; since these infections require different treatment regimens and often have relapses, which require long-term management.

Considering the geographical coincidence and the magnitude of these threats, *An. stephensi* is clearly not an isolated biological challenge; and not necessarily the most important in so far as malaria control and elimination in affected countries is concerned. Efforts to address biological challenges should therefore be integrated and resources directed appropriately to suit the different contexts.

## Understanding and responding to the threat

### Role of Anopheles stephensi in the transmission of malaria in Africa

Malaria transmission systems can be complex, often involving multiple vector species and in some cases multiple *Plasmodium* species circulating. The importance of any vector species in pathogen transmission depends on multiple factors, including occurrence and biting densities, host preferences, survival strategies, and proclivities for biting either indoors or outdoors. Equally important is the differential species-level competence, as defined by the ability of the mosquitoes to pick up *Plasmodium* gametocytes from an infected human, mature these parasites through multiple stages in their gut, and eventually transmit the infective sporozoites to a susceptible human. To identify the most dominant vector species in an area, it is therefore important to assess not just their densities and biting patterns, but also their proportional contribution to overall transmission events relative to other species. This requires a broad assessment of the prevalence of *Plasmodium* sporozoites in populations of multiple *Anopheles* species co-occurring in an area, then computing and comparing the entomological inoculation rates (EIRs) associated with each one.

Previous reports have indicated that *An. stephensi* was plausibly associated with malaria epidemics [1, 18]. However, a recent meeting of experts concluded that further assessments are necessary to establish the degree to which the species actually contributes to malaria epidemiology in the affected areas [10]. Based on such local data, controlling invasive species such as *An. stephensi*, should be done in ways that do not ignore the other important species in the area. Efforts must be made to assess the contribution of the species to overall malaria transmission and to understand the potential ecosystem factors that may increase its competence.

Most field studies on *An. stephensi* have been in Asia, where the species contributes to significant proportions of the malaria cases, even though other species such as *Anopheles culicifacies, Anopheles fluviatilis* and *Anopheles minimus* appear more dominant. In the Horn of Africa region, *An. stephensi* has been found to co-exist with other *Anopheles* species, mostly *An*. *arabiensis* in the same localities [19] (and its aquatic forms co-occupy the same habitats as *Aedes* mosquitoes [8]). Both *An. arabiensis* and *An. stephensi* species exhibit indoor-biting and outdoor-biting behaviors, and can readily blood-feed on non-human hosts [20, 21]. The lack of *Plasmodium* infections in the *An. stephensi* samples from eastern Ethiopia, despite being an area of low malaria transmission, may suggest comparatively low competence of the species in such settings [19]. A more recent update using data from 2019 also reported no *P. falciparum* infections in field-collected *An. stephensi*, though a small number of the mosquitoes (3 out of 780) were positive for *P. vivax* [22]. This should be investigated further, especially since laboratory tests in Ethiopia have demonstrated that *An. stephensi* can have higher susceptibility to malaria infections than *An*. *arabiensis* [23]. In all affected countries, it will be important to validate such findings using expanded field surveys estimating the direct contribution to local EIR estimates. Such surveys should also include investigations into the breeding, feeding and resting behaviours of vector species. Fortunately, there are ongoing studies, which could eventually answer these important questions [10].

Host preferences of individual mosquito species play a significant role in the contribution of that species to malaria transmission [24]. Studies in Asia and Africa have shown that *An. stephensi* are strongly zoophilic, with human blood indices (HBI) sometime as low as 0.009, indicating very low propensities to bite humans [25]. From a series of 37 multi-country field observations, the mean HBI for *An. stephensi* was previously estimated to be 0.023 [26]. In contrast, far higher HBIs were recorded for the other Afro-tropical malaria vectors, *An. gambiae* (estimated mean HBI = 0.93; 36 field observations), *An. funestus* (0.98; 30 field observations) and *An. arabiensis* (0.87; 32 field observations) [26]. In a recent survey in Ethiopia, 631 blood-fed *An. stephensi* collected from multiple resting surfaces in human and animal shelters in Ethiopia were screened, and only one of them was found with human blood, the rest having fed on other vertebrates [22]. The degree of anthropophily is a reflection of multiple intrinsic and extrinsic factors, perhaps the most important being the proximal availability of the different vertebrate host species across geographies [24]. Despite the plasticity of these relationships, the high HBIs in the other vector species is associated with the stability of malaria in sub-Saharan Africa [26, 27].

On the basis of the reported low HBIs in *An*. *stephensi*, we question whether this species is indeed as big of a threat to malaria control in Africa as it is being projected. It is unlikely that this vector alone could sustain high malaria transmission rates in multiple locations; but the species may play a significant role in local settings and in sporadic outbreaks, particularly in urban areas, as was recorded in Djibouti [1, 18, 28]. The outbreak in Djibouti was itself compounded with challenges to implement malaria vector control interventions, and could not be attributed entirely to the presence of *An. stephensi*. In these specific settings, ITNs and IRS can be unsuccessful due to poor access to the human populations. Despite this assertion, any detection of *An. stephensi* in any urban areas or in low transmission settings should trigger greater vigilance and response to prevent possible outbreaks. While there are no such examples in the African continent, *An. stephensi* has recently been detected in Sri Lanka [29], which already eliminated malaria in 2016 [30].

Additionally, the biting behaviors and the subsequent low HBIs in this species suggest that indoor vector control methods such as ITNs may not be the most appropriate. This emphasizes the need for an expanded set of approaches, which may include integrated larval source management programs targeting both *Aedes* and *An. stephensi* mosquitoes.

### Plausible presence and spread of An. stephensi beyond the Horn of Africa region

Identifying the origin of current *An*. *stephensi* populations spreading in Africa has been an important challenge for researchers. Phylogenetic analysis of samples collected in the Horn of Africa suggest it may have originated in southern Asia and the Arabian Peninsula. Studies in Ethiopia have found evidence of high genetic diversity and geographical structuring, and concluded that the populations found in south-eastern parts of the country were more recent than those in the north-eastern areas [31]. This report, together with an earlier analysis concluded that *An. stephensi* populations in this region were closely linked to samples from Pakistan, and therefore most likely originated from south Asia as opposed to the Arabian Peninsula [2, 31]. In eastern Ethiopia, even samples collected far inland were often found along transport routes [19], suggesting that the geographical dispersal may be driven by human activities and that there may be a common origin.

The general assumption is therefore that the species was brought through ships from India and Pakistan. However, as these ships dock along several other Indian Ocean and Red Sea ports, it is reasonable to expect or anticipate independent introductions of *An. stephensi* in and around other coastal cities such as Lamu and Mombasa in Kenya, Tanga, Dar es Salaam, Zanzibar and Mtwara in Tanzania, Maputo in Mozambique and even Durban in South Africa. An important question therefore is why *An*. *stephensi* has not yet been reported in these other areas. It will be important to enhance surveillance in these localities to determine if the species is present.

Beyond these introductions, it is clear that *An. stephensi* is rapidly spreading within Africa. Though this data is limited to national borders, the extensive geographical spread in Ethiopia [19, 31] and Sudan [5] are evidence that the vector species could already have domiciled in many sites distant from the initial port of entry. In the case of Sudan, where *An. stephensi* has been found far inland in precincts bordering six countries without any previous sightings including the landlocked Chad, Central African Republic and South Sudan [5]. It can be argued therefore that it is only a matter of time before the vector species is found in wider geographies beyond the Horn of Africa, far into the hinterland of Africa.

### Anopheles stephensi in urban and rural areas

*Anopheles stephensi* exploits several larval habitat types—ranging from human-made water containers such as plastic tanks, cisterns, barrels, discarded tyres and plastic containers, to freshwater pools such as the margins of water streams and in irrigation ditches [1, 2, 18, 19, 29, 32]. Due to sub-optimal urban planning and insufficient provision of piped water, there is often a tendency of urban residents to store containerized water for home use, thus favoring the breeding of *An*. *stephensi*. It is therefore not surprising that the species is often found in the same habitats as *Ae. aegypti* – an important vector of dengue in Africa in urban areas [19]. Moreover, solid waste management is a common challenge in such settings, further expanding opportunities for these vectors to breed.

Because of continuing expansion of urban centers and the changing structure of housing in rural and urban areas, it is more appropriate to define the rural-urban transitions on a continuum rather than as distinct categories. Similarly, while *An. stephensi* is mostly an urban vector, the species may extend its ecological extents into more rural areas where habitats like streams and irrigation ditches abound. This would further complicate the challenges related to identification and reporting of the vector species beyond the African coastal cities. Moreover, the degree of such extra-urban extension is hard to predict and will require additional investigations to understand the vector behaviors and trajectories.

### Enhancing surveillance-response systems

The concerns regarding increased risk of urban malaria mediated by *An*. *stephensi* should be underscored [6]. However, the best results will be achieved by broadly strengthening the surveillance-response initiatives in both rural and urban settings, with the specific efforts targeting *An. stephensi* included within those initiatives. This way, authorities can update the available data based on any new sightings of different vector species. For all at-risk areas, such as those previously predicted [7], precautionary surveillance efforts should be incorporated into existing programs to increase the likelihood of detecting *An. stephensi* incase it is already present or spreading. As already shown in Sri Lanka [32], there is also an urgent need to monitor anthropogenic factors associated with possible invasion and expansion, particularly in locations predicted to potentially harbor the vector [7].

Given the current gaps in vector surveillance, there is need to carefully re-examine the history and evolution of entomological surveillance practices in sub-Saharan Africa. The close morphological similarity of *An*. *stephensi* to other *Anopheles* species such as *An. arabiensis*, and the historical absence of molecular tools—incorporating both Polymerase Chain Reaction (PCR) and sequencing for identification, may explain why the former species could have been missed. Indeed, during the late 70’s and early 80’s, the focus was on identifying members of the *An*. *gambiae* group of species using polytene chromosomes and iso-enzymes [33–35]. While none of these tools could have picked up *An. stephensi*, it was a lost opportunity which needed a change of mindset—appreciating that what looked morphologically like members of the *An*. *gambiae* complex could have been something else. Fortunately, recent updates in the main taxonomic keys for African *Anopheles* mosquitoes now include *An. stephensi* [36] and could be readily incorporated to expand effective surveillance responses in at-risk areas. Even the most experienced field biologists conducting surveys should consider regularly going back to the basics – to use the morphological keys for species identification.

Certain parallels can be drawn between the expanding range of *An. stephensi* in the Horn of Africa and the 1930s human-mediated invasion by *An. gambiae* s.l. in Brazil [37, 38]. Here, the sibling species of concern, more recently identified to be *An. arabiensis* based on museum specimen [39], was able to spread extensively, covering an area of 54,000 Km^2^ (more than two times larger than the countries of Djibouti or Togo), in less than a decade. This heralded a major public health disaster before it was rounded up and contained through integrated efforts primarily targeting aquatic habitats and human habitations with insecticide treatments [37, 38]. It is unclear what the current geographical extent of *An. stephensi* is, or whether there have been multiple other introductions. However, the lessons from the Brazil campaign suggest that focused attention to surveillance and control relying on the basic understanding of the vector biology could be more important than waiting for sophisticated laboratory research [37]. The plans by stakeholders in Ethiopia to implement integrated surveillance and control of *An. stephensi* and *Aedes aegypti*, with targeted elimination of the former (Dr. Fitsum Girma Tadesse, Personal Communication) are therefore commendable and could provide a blueprint for the region. While this section has focused mostly on entomological surveillance, it is recognized that other aspects of malaria surveillance must also be strengthened, notably epidemiological surveillance, both inland and at ports of entry.

### Engaging different stakeholder groups to support vector surveillance and control

Many of the factors associated with invasion and spread of *An. stephensi* in Africa are beyond the scope of a typical health ministry or department. To ensure sustained and effective responses, authorities must build partnerships between multiple sectors, e.g. environment, health, education, local government/councils, trade and industry, agriculture, housing and financing. There should also be sufficient integration within sectors, one example being the need to join key surveillance response initiatives for *Anopheles* and *Aedes*-borne diseases. One example of a specific focus area for such integration may be around search and control operations targeting the vectors in their aquatic stages. In this case, the environmental management and sanitation agencies can be particularly important for improving waste management and reducing mosquito breeding. On the other hand, the ministries of education can support both public and school-based education programs that empower communities to identify and address risk factors associated with the vectors. Similarly, individuals, households and leaders in affected communities will also play a critical role in interventions targeting *An. stephensi* and other emergent threats. For example, promotion of household and community-level activities including covering water storage containers, removing standing water, and use of larvicides in storage containers could be considered.

## Conclusions

While the presence of *An. stephensi* in Africa is a concern, it should be viewed as a symptom of a broader and more important problem—weakened vector surveillance and control programs. The invasion and spread of this vector species is one of multiple biological threats with potential to reverse malaria control and elimination efforts. Research efforts should establish relative contributions of *An. stephensi* in different malaria transmission settings and identify important ecological and anthropogenic determinants of the species’ spread to inform control. Countries and partners should enhance their surveillance systems to map the geographical extents and trajectories of the vectors in and beyond urban areas; and create integrated control programs operating across key sectors and on multiple vector species. The programs should resist any temptation to focus on *An. stephensi* as a stand-alone threat, but instead adopt more holistic and integrated approaches. Lastly, it is important to involve local stakeholders in affected communities, and to tailor programs to the local context.

## Data Availability

Not applicable.
